# Responses of Intestinal Mucosal Barrier Functions of Rats to Simulated Weightlessness

**DOI:** 10.3389/fphys.2018.00729

**Published:** 2018-06-14

**Authors:** Mingliang Jin, Hao Zhang, Ke Zhao, Chunlan Xu, Dongyan Shao, Qingsheng Huang, Junling Shi, Hui Yang

**Affiliations:** ^1^Key Laboratory for Space Bioscience and Biotechnology, School of Life Sciences, Northwestern Polytechnical University, Xi’an, China; ^2^College of Food Engineering and Nutritional Science, Shaanxi Normal University, Xi’an, China

**Keywords:** simulated weightlessness, intestine, mucosal barrier, microbiota, TLR4/MyD88/NF-κB signaling pathway

## Abstract

Exposure to microgravity or weightlessness leads to various adaptive and pathophysiological alterations in digestive structures and physiology. The current study was carried out to investigate responses of intestinal mucosal barrier functions to simulated weightlessness, by using the hindlimb unloading rats model. Compared with normal controls, simulated weightlessness damaged the intestinal villi and structural integrity of tight junctions, up-regulated the expression of pro-apoptotic protein Bax while down-regulated the expression of anti-apoptotic protein Bcl-2, thus improved the intestinal permeability. It could also influence intestinal microbiota composition with the expansion of Bacteroidetes and decrease of Firmicutes. The predicted metagenomic analysis emphasized significant dysbiosis associated differences in genes involved in membrane transport, cofactors and vitamins metabolism, energy metabolism, and genetic information processing. Moreover, simulated weightlessness could modify the intestinal immune status characterized by the increase of proinflammatory cytokines, decrease of secretory immunoglobulin A, and activation of TLR4/MyD88/NF-κB signaling pathway in ileum. These results indicate the simulated weightlessness disrupts intestinal mucosal barrier functions in animal model. The data also emphasize the necessity of monitoring and regulating astronauts’ intestinal health during real space flights to prevent breakdowns in intestinal homeostasis of crewmembers.

## Introduction

Exposure to microgravity or weightlessness lead to alternations in multiple systems, such as cephalad fluid shift, space motion sickness, muscle atrophy, bone demineralization, and immune system dysregulation. It could also cause various adaptive and pathophysiological alterations in digestive structures and physiology, thus induce slowdown of nutrient digestion and absorption, and disorder of intestinal immunity (Rabot et al., 2000). For instance, reduced length of intestinal villi, decreased mucin production of intestinal epithelial cells was found in rats after space flights missions (Shi et al., 2017). A significantly higher relative expression of colonic transforming growth factor-β (TGF-β), which contributes to epithelial migration and wound healing, was observed in flight mice flown on STS-135 for 13 days (Ritchie et al., 2015). Previous studies have demonstrated that the hindlimb unloading, a well-accepted ground-based spaceflight analog, can impair the integrity of intestine and reduce its ability against bacterial infection (Belay et al., 2002). It indicated that during spaceflight the intestine encounters increased risk for microbial infection (Cervantes and Hong, 2016). The microgravity and related low fluid shear dynamics has also been proved to be able to regulate microbial gene expression, physiology and pathogenesis (Horneck et al., 2010). Certain bacteria obtain increased pathogenic features after exposure to microgravity or spaceflight, including improved growth rate and virulence, enhanced resistance to several environmental stress, altered responses to antibiotics and increased biofilm formation (Li et al., 2015; Liu, 2017; Shi et al., 2017). Those may heighten the risk for intestinal microbial infections mentioned above.

In recent years, there has been a renewed interest concerning that intestinal microbiota may be influenced by environmental and genetic factors, diet and probiotics, which also affect the host health and behavior in turn (Faust and Raes, 2016). Compositional changes in intestinal mucosal microbiota have been reported during short and long-term spaceflight, which indicated that microgravity could perturb the structure and composition of intestinal bacterial communities (Nefedov et al., 1971; Smirnov and Lizko, 1987; Ritchie et al., 2015). However, studies on how commensal bacteria respond to weightlessness at species level and the influence of weightlessness on their functionality and metabolic activities are limited.

The intestinal mucosa acts as a barrier to harmful substances (Chen et al., 2013), thus maintaining the intestinal intracellular homeostasis. The disruption of intestinal mucosal barrier functions, including dysbiosis and dysfunction of intestinal immune system, is closely associated with pathogenesis of various intestinal diseases (Li et al., 2015). The present study was conducted to investigate the response of intestinal barrier functions of rats to hindlimb unloading, a well-accepted weightlessness analog. In detail, the structure of intestine was observed; the expressions of apoptosis-associated proteins and tight junction-related proteins in ileum were analyzed; the levels of several serum inflammatory cytokines and peripheral blood cells were detected. Furthermore, the change of TLRs/MyD88/NF-κB signaling pathway in response to hindlimb unloading was investigated. More importantly, the influence of gut microbiota under simulated weightlessness at species level was studied, the bacterial metagenomes of gut microbiota were predicted from 16S rRNA data, and categorized by function with Kyoto Encyclopedia of Genes and Genomes (KEGG) ontology.

## Materials and Methods

### Animal Model and Experimental Design

Twenty male Sprague-Dawlery (SD) rats, weighting 199 ± 15.7 g, were purchased from Experimental Animal Center, College of Medicine, Xi’an Jiaotong University. All rats, according to weight, were randomly divided into two groups with ten each: the normal control group (CON) and the hindlimb unloading group (SUS). The hindlimb unloading model was established as described previously (Zhou et al., 2012; Li et al., 2015). Briefly, each rat was suspended by the tail at approximately 30 degrees head-down tilt without load bearing on the hindlimbs for 21 days. The animals were housed in plastic cages individually at room temperature (22 ± 1°C) under a 12 h light-dark cycle, and provided with commercial pellet diet and water *ad libitum* by use of water bottles and food distributed around the floor of the cage. Animals demonstrated no adverse effects or pronounced weight loss. At the end of the experiment (21 days), the animals were feed-deprived overnight, and anesthetized with diethyl ether. Peripheral blood samples were obtained by inferior vena cava puncture. About 1 ml of blood samples were collected into a vacuum tube containing EDTA-K_2_ anticoagulant for blood cell counts, and the rest were centrifuged at 1000 × *g* for 10 min at 4°C for serum. Ileums were immediately excised, washed with ice-cold saline, and blotted dry. Caecal contents were collected and stored in freezing tubes. All of the samples were frozen by immersion in liquid nitrogen and stored at -80°C. The experiments were approved by the Institutional Animal Care and Use Committee of Northwestern Polytechnical University, and performed in accordance with the institutional ethical guideline of experimental animals.

### Intestinal Morphology and Immunohistochemistry

The collected duodenum, jejunum and ileum were washed with normal saline immediately, then fixed in 4% neutral-buffered formalin, dehydrated, and embedded in paraffin. The tissues were consecutively cut into 4-μm thick sections, and stained with hematoxylin and eosin (HE). The fluorescence microscope (Nikon, Japan) was used to observe the intestinal morphology.

The paraffin sections of ileum were used for immunohistochemistry analysis, and the staining was performed according to previous report (Shan et al., 2016). The primary antibodies used were the following: rabbit polyclonal Bax and mouse monoclonal Bcl-2 (GB11007 and GB12008, Servicebio, China), rabbit polyclonal claudin-1 (No. 4399, Cell Signaling Technology, China) and rabbit polyclonal E-cadherin (AF0131, Affinity Biosciences, China). Horseradish peroxidase (HRP)-conjugated goat anti-rabbit/mouse IgG was used as the secondary antibody. All images were captured using the fluorescence microscope (Nikon, Japan).

### ELISA

About 0.5 g of ileum was minced and homogenized (10%, w/v) in normal saline solution. The homogenate was centrifuged at 1700 × *g* for 10 min at 4°C, and the resulting supernatant fractions were collected. The levels of secretory immunoglobulin A (SIgA) in ileum and the concentrations of interferon-γ (IFN-γ), interleukin-2 (IL-2), IL-4, diamine oxidase (DAO) and endotoxin (ET) in serum were detected with Synergy HT Multi-Detection Microplate Reader (Bio-Tek) using corresponding commercial ELISA kits (R&D System, Minneapolis, MN, United States).

### Western Blotting

Western blotting was performed according to our previous report (Jin et al., 2017). Briefly, the ileums from 3 randomly selected rats of each group were homogenized in radioimmunoprecipitation assay (RIPA) buffer containing fresh protease inhibitor mixture (Sangon Biotech, China). The total protein concentration in the supernatant was measured by the bicinchoninic acid (BCA) protein assay kit (Bio-Rad, Hercules, CA, United States). The proteins from each sample were separated by 15% sodium dodecyl sulfate-polyacrylamide gel electrophoresis (SDS–PAGE) followed by transfer onto polyvinylidene difluoride membranes (PVDF, Millipore, Billerica, MA, United States). After being blocked in 5% non-fat milk solution, the membranes were incubated with primary antibodies at 4°C overnight, followed by secondary incubation with HRP-conjugated anti-rabbit/mouse IgG (BA1003, 1:100; Boster, China) at room temperature for 30 min. The following primary antibodies were used: rabbit polyclonal claudin-1 (No. 4399, 1:1000; Cell Signaling Technology, China), rabbit polyclonal claudin-5 (bs-10296R, 1:1000; Bioss Antibodies, China), rabbit polyclonal E-cadherin (AF0131, 1:1000; Affinity Biosciences, China), rabbit polyclonal occludin and myeloid differentiation factor 88 (MyD88) (ab31721 and ab2064, 1:1000; Abcam, United States), rabbit polyclonal toll-like receptors 4 (TLR4) (BA1717, 1:400; Boster, China), rabbit monoclonal inhibitor of κB (IκB) (No. 1130, 1:1000; Epitomics, China), mouse monoclonal nuclear factor-κB (NF-κB) p65 and β-action (sc-8008 1:500; sc-1616r, 1:1000; Santa Cruz, United States). Immunoreactive bands were visualized by enhanced chemiluminescence (Millipore, Billerica, United States) reaction, and quantified by intensities using AlphaEaseFC software (Alpha Innotech, San Leandro, CA, United States). The relative expression levels of proteins were expressed as the gray value of the target band over the gray value of β-actin in the same sample.

### Peripheral Blood Cell Analysis

The concentration of white blood cells (WBC), and the relative percentage of neutrophil (NEU), lymphocyte (LYM), and monocyte (MON) in anticoagulated blood samples were determined using an automated hematology analyzer (Sysmex 2100, Sysmex, Kobe, Japan).

### DNA Extraction, PCR Amplification and Pyrosequencing

Bacterial total genomic DNA from each cecal content samples was extracted using the E.Z.N.A^®^ Genomic DNA Isolation Kit (Omega Bio-Tek). The V1-V3 regions of the bacterial 16S rRNA gene were amplified by PCR (95°C for 2 min, followed by 25 cycles at 95°C for 30 s, 55°C for 30 s, 72°C for 30 s and a final extension at 72°C for 5 min) using universal eubacterial primers 27F (5′-AGAGTTTGATCCTGGCTCAG-3′) and 533R (5′-TTACCGCGGCTGCTGGCAC-3′) on GeneAmp^®^ PCR System (9700, Applied Biosystems, Singapore). A ten-base barcode sequence unique to each sample was designed in these primers so that multiple samples could be analyzed in a single sequencing run (Jin et al., 2017). The PCR reactions were performed in a triplicate 20 μl mixture containing 4 μl of 5 × Fastpfu reaction buffer, 250 μM of dNTPs, 0.2 μM of each primer, 10 ng of template DNA, and 2.5 U of Fastpfu DNA Polymerase (TransGen Biotech, Beijing, China). Then amplicons of the same samples were pooled, checked by 2% agarose gel electrophoresis, purified using the AxyPrep DNA Gel Extraction Kit (Axygen Biosciences, China), and quantified using QuantiFluor^TM^ double-stranded DNA System (Promega, United States). Purified amplicons from each reaction mixture were pooled in equimolar ratios based on concentrations, and then subjected to emulsion PCR using Roche GS FLX Titanium emPCR Kit to generate amplicon libraries according to the standard protocols from 454 Life Sciences. The pyrosequencing was performed on Roche Genome Sequencer GS FLX Titanium platform at Shanghai Majorbio Bio-Pharm Technology Co., Ltd., Shanghai, China. The sequence data were uploaded in the NCBI Sequence Read Archive with the accession number SRP148837 under BioProject No. PRJNA472839.

### Bioinformatics and Statistical Measurements

The pyrosequencing data was analyzed by the quantitative insights into microbial ecology (QIIME) v.1.9.1 software package (Caporaso et al., 2010b) according to our previous report (Jin et al., 2017). Briefly, sequences were demultiplexed and assigned to individual samples according to the specific barcode. Barcodes and primers were trimmed, with exact barcode matching and no nucleotide mismatch in primer matching. Sequences shorter than 100 bp or longer than 1000 bp, having one or more ambiguous base, or with an average quality score less than 20 were removed from the data set. The chimeras were identified using the UCHIME method (Edgar, 2010) against the GOLD database and discarded from further analyses. Operational taxonomic units (OTUs) were clustered with a 97% similarity threshold using USEARCH (Edgar, 2010). The most abundant sequence was selected as the OTU representative, and aligned against the core set of Greengenes 13.5 (DeSantis et al., 2006) using PYNAST (Caporaso et al., 2010a) with a minimum length of 150 bp and a minimum percent identity of 75.0%. A 16S alignment Lane mask supplied by QIIME was used to remove hypervariable regions from the aligned sequences. OTUs were taxonomically classified using the ribosomal database project (RDP) classifier with a standard minimum support threshold of 80% (Wang et al., 2007). For unidentified OTUs, closest hits using BLAST against NCBI 16S rRNA database were cross referenced with >90% query cover, >80% identity, and <0.001*E*-value. Community diversity was assessed with rarefaction analysis, Chao 1, Shannon and Simpson indexes (Mao et al., 2015). Principal coordinates analysis (PCoA) from QIIME output was produced based on unweighted UniFrac distance (Lozupone and Knight, 2005). Differentially abundant taxa were identified using the linear discriminant analysis (LDA) effect size (LEfSe) method^1^ (Segata et al., 2011) at the genus and OTU levels following the protocol described in the previous study (Zhang et al., 2013). Briefly, the non-parametric factorial Kruskal–Wallis test (α = 0.05) was used to analyze the differences between treatments, with the threshold on the logarithmic LDA score for discriminative features more than 2.0.

The molecular functions or metagenomes of intestinal microorganisms were predicted from marker genes, in this case 16S rRNA by using Phylogenetic Investigation of Communities by Reconstruction of Unobserved States (PICRUSt) (Langille et al., 2013). Close reference OTU picking against the Greengens 13.5 reference taxonomy, with OTUs assigned at 97% identity, was used to generate BIOM-formatted OTU table in QIIME (Mao et al., 2015). The 16S copy number was normalized, metagenome was predicted and molecular functions were categorized into KEGG pathways on the web-based Galaxy^2^ according to the instructions described by the developers^3^. The LEfSe algorithm was used to identify specific functions and characterize the differences between two groups according to the previous report (Vázquez-Castellanos et al., 2015).

Differences in cytokines levels, proteins expression and blood cells between two groups were evaluated by both parametric (Student’s *t*-test) and non-parametric (Mann–Whitney *U*-test) methods using GraphPad Prism software (Version 6.0c, GraphPad, La Jolla, CA, United States).

## Results

### Intestinal Mucosal Morphology

**FIGURE 1:**
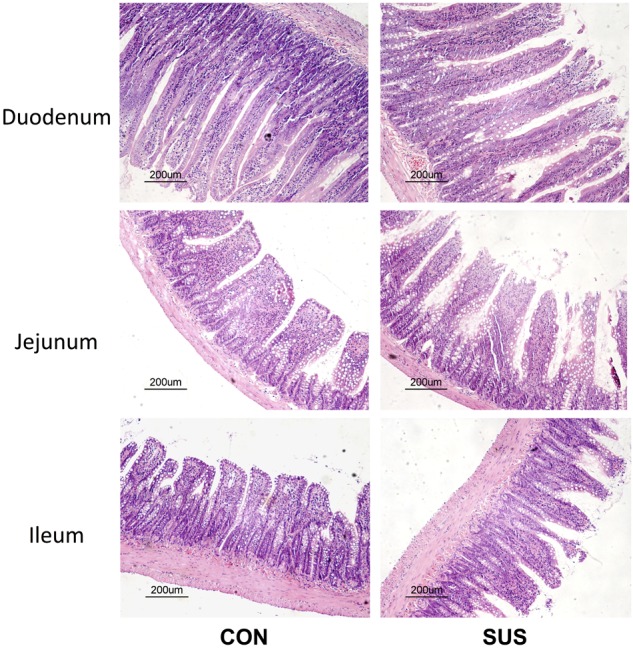
Effects of simulated weightlessness on intestinal morphology in rats. CON, control group; SUS, simulated weightlessness group.

To evaluate the effects of simulated weightlessness on intestinal mucosal morphology, HE-stained duodenum, jejunum and ileum sections were examined by light microscopy (**Figure 1**). Histologic analysis indicated a normal pattern in CON group. The intestinal villi were packed and intact. In contrast, the intestinal mucosa from rats in SUS group showed conspicuous morphological changes. Observation of duodenum, jejunum and ileum revealed destruction of microvilli, submucosal vasodilation and congestion in SUS group. The intestinal villi became erosive, hyperemic, with inflammatory cells among villous stroma. In addition, the intestinal mucosa showed necrosis, exfoliation and signs of atrophy with widened tight junctions. Immunohistochemistry staining indicated that the protein expression of Bax was significantly up-regulated, while Bcl-2 protein expression was down-regulated in ileum following structural destruction in SUS group (**Supplementary Figure S1**).

### Expression of Tight Junction-Related Proteins and TLRs/MyD88/NF-κB-Associated Proteins in Ileum

**FIGURE 2:**
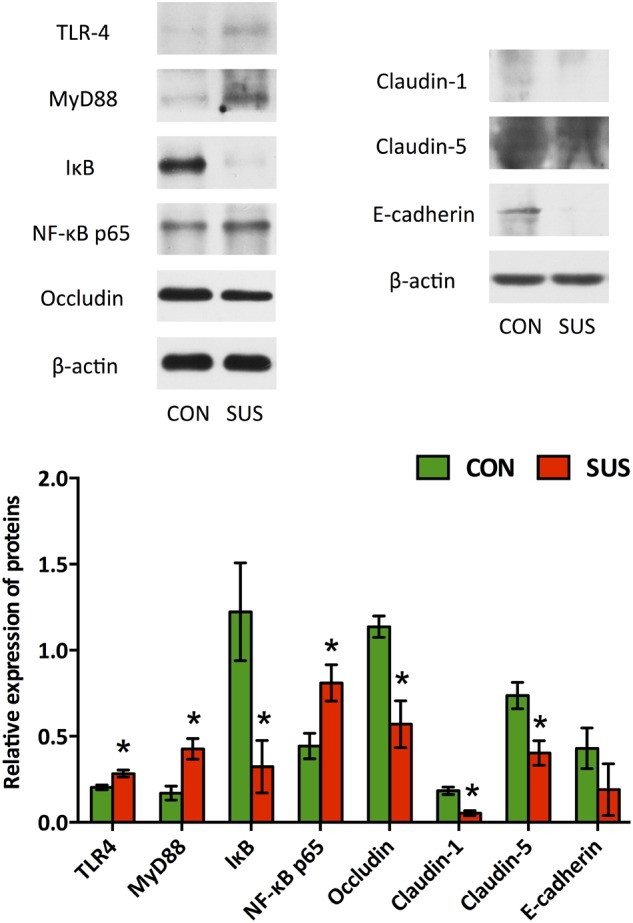
Effects of simulated weightlessness on the expressions of occludin, claudin-1, claudin-5, TLR-4, MyD88, IκB and NF-κB p65 in ileum. CON, control group; SUS, simulated weightlessness group. Data are representative as means ± SEM. ^∗^*P* < 0.05, compared with the control group.

Western blotting analysis (**Figure 2**) showed that the expressions of occludin, claudin-1, claudin-5 and IκB dramatically decreased (*P* < 0.05) when the rats were tail-suspended for 21 days. Immunohistochemistry analysis also indicated that tail-suspension induced a significantly decrease in expression of claudin-1 and E-cadherin, which mainly localized to the apical surface of intestinal villi and crypt in ileum (**Supplementary Figure S2**). In contrast, the expression of TLR-4, MyD88 and NF-κB p65 were significantly up-regulated by tail-suspension in ileum (*P* < 0.05).

### Levels of Cytokines, DAO and ET in Serum and SIgA in Ileum

**FIGURE 3:**
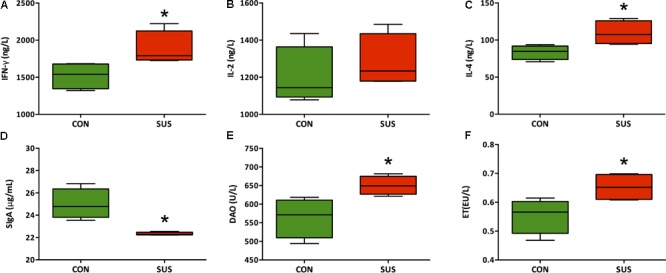
**(A–F)** Effects of simulated weightlessness on the levels of IFN-γ, IL-2, IL-4, DAO, and ET in serum and SIgA in ileum. CON, control group; SUS, simulated weightlessness group. Data are representative as median with absolute range (min. and max.) and interquartile ranges Q25 and Q75. ^∗^*P* < 0.05, compared with the control group.

As shown in **Figure 3**, hindlimb unloading for 21 days significantly increased serum concentrations of IFN-γ, IL-4, DAO and ET (*P* < 0.05), whereas significantly decreased the level of SIgA in ileum (*P* < 0.05). The difference of serum level of IL-2 was not significant between two groups (1200 ± 79.9 ng/L vs. 1282 ± 71.7 ng/L, *P* > 0.05).

### Peripheral Blood Cell Count

**FIGURE 4:**
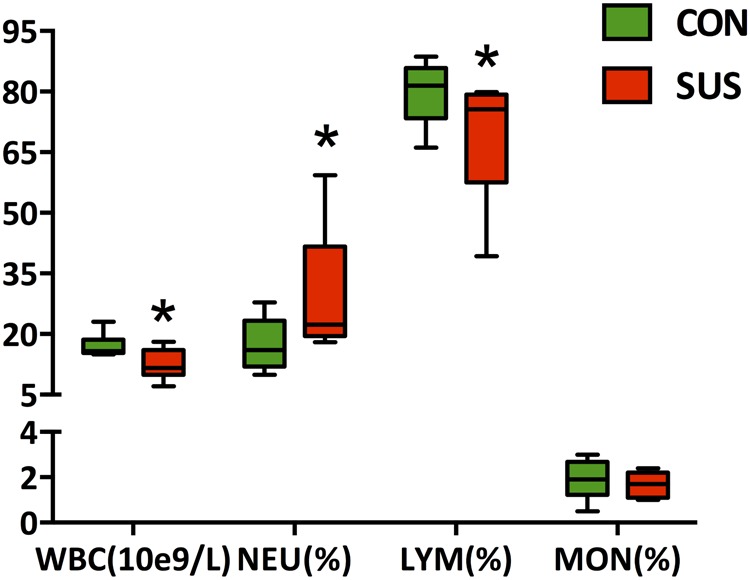
Effects of simulated weightlessness on the concentration of WBC, and the relative percentage of NEU, LYM, and MON in peripheral blood. CON, control group; SUS, simulated weightlessness group. Data are representative as median with absolute range (min. and max.) and interquartile ranges Q25 and Q75. ^∗^*P* < 0.05, compared with the control group. WBC, white blood cells; NEU, neutrophils; LYM, lymphocytes; MON, monocytes.

The hematological analyses (**Figure 4**) showed that hindlimb unloading for 21 days significantly decreased the concentration of WBC and LYM (*P* < 0.05), while markedly increased the relative percentage of NEU (*P* < 0.05) in peripheral blood compared with the control rats.

### Overall Structure of Intestinal Microbiota

**FIGURE 5:**
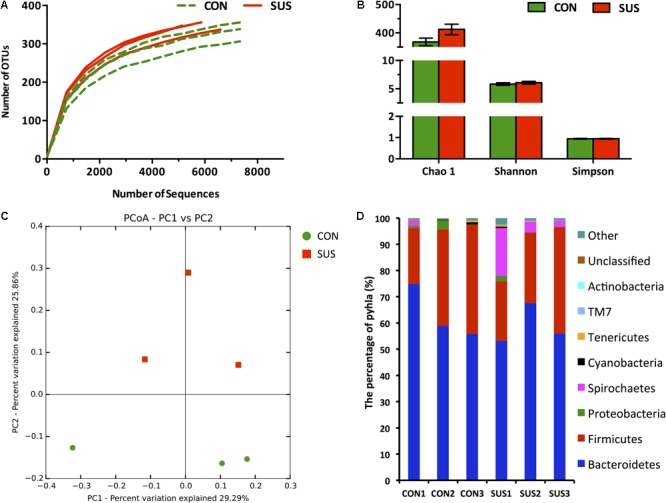
Pyrosequencing analysis of caecal bacteria. **(A)** Rarefaction curves. **(B)** Diversity indexes. Values are representative as means ± SEM. **(C)** Principal coordinates analysis of caecal bacteria at the OTU level. **(D)** Phylum-level abundance of 16S rRNA gene sequences from the cecum. CON, control group; SUS, simulated weightlessness group.

A total of 59254 qualified raw read were obtained from 6 caecal content samples with an average of 9876 ± 1058 reads per sample using the 16S rRNA sequencing method. 701 OTUs were detected (346 ± 20 per sample) based on a 97% nucleotide sequence identity. Individually based rarefaction curves showed that the observed OTUs almost reached the plateau (**Figure 5A**). There was no significant difference in Chao 1, Shannon and Simpson indexes between CON and SUS groups (**Figure 5B**). Moreover, Unifrac and OTU-based PCoA, represented as a discrete data point without overlap, revealed distinct clustering of samples from CON and SUS groups and distinct microbiota composition between two groups (**Figure 5C**).

### Comparison of Intestinal Bacteria

The RDP classifier was used to taxonomically assign OTUs from phylum to genus levels at 80% confidence threshold. Among the bacterial groups, Bacteroidetes and Firmicutes were the 2 predominant phyla, contributing 63.2 and 33.2% of gut microbiota in the CON group, and 58.9 and 30.1% in the SUS group, respectively (**Figure 5D**). Spirochaetes and Proteobacteria constituted the next 2 dominant phyla at 0.66 and 1.52% in CON rats and 8.20 and 0.83% in SUS rats, respectively. Cyanobacteria, Tenericutes, TM7 and Actinobacteria, with the total amount of 0.80% in CON group and 0.63% in SUS group, were also detected in the caecal content samples.

**FIGURE 6:**
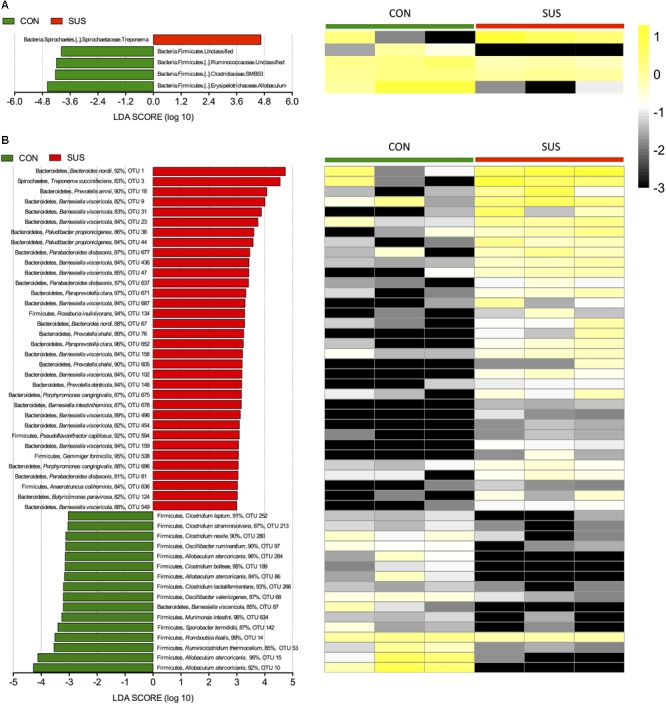
Comparisons of caecal bacteria using LEfSe. The left histogram shows the LDA scores computed for features at the phylum **(A)** and OTU level **(B)**. The right heatmap shows the relative abundance of phylum and OTU (log 10 transformed). Each column represents one animal and each row represents the phylum and OTU corresponding to left one. CON, control group; SUS, simulated weightlessness group.

LEfSe was employed to identify specific phylotypes responding to 21 days of hindlimb unloading. At the genus level, the relative abundance of *Treponema* in *Spirochaetes* phylum was significantly higher (LDA score > 2), while four genera including *Allobaculum, SMB53* and unclassified Ruminococcaceae in *Firmicutes* phylum were significantly lower (LDA score > 2) in SUS group compared with CON group (**Figure 6**). At the OTU level, 50 key phylotypes were discovered as high-dimensional biomarkers for separating gut microbiota between CON and SUS rats, among which, 34 were significantly increased, and 16 were significantly decreased (LDA score > 3) in SUS group compared with CON group (**Figure 6B**). Of the 34 increased OTUs, 29 belonged to the phylum Bacteroidetes, and of the 16 decreased OTUs, 15 belonged to the phylum Firmicutes. At the species level, many were significantly enriched in the SUS group, including *Bacteroides nordii, Treponema succinifaciens, Paludibacter propionicigenes, Parabacteroides distasonis, Paraprevotella clara, Porphyromonas cangingivalis, Barnesiella intestinihominis* and three *Prevotella* species, while some were significantly decreased, including *Allobaculum stercoricanis, Ruminiclostridium thermocellum, Romboutsia ilealis, Sporobacter termitidis, Murimonas intestine*, two *Oscillibacter* species and four *Clostridium* species. Overall, trends showed that Bacteroidetes OTUs were increased, and Firmicutes OTUs were decreased in the SUS groups, leading to an overall lowering of the average Firmicutes-to-Bacteroidetes (F:B) ratio in SUS group compared with CON group (0.517 vs. 0.551, *P* > 0.05).

### Predicted Molecular Functions of Intestinal Microbiota

**FIGURE 7:**
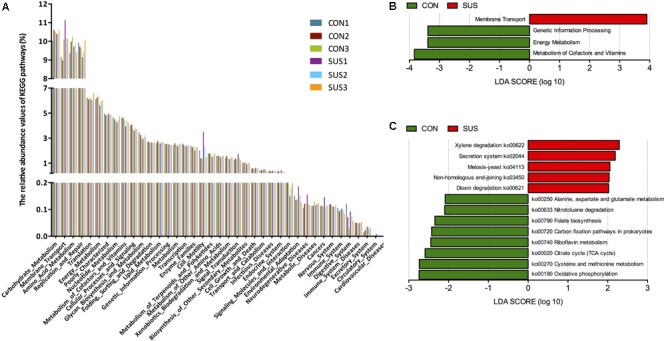
Metagenomic functional predications associated to the microbiota profiles. **(A)** Relative abundance of the predicated gene of metagenome related to KEGG pathway at level 2. **(B)** and **(C)** Predicated genes of metagenome related to KEGG pathways and KEGG orthology differentially represented between two groups identified by LEfSe. CON, control group; SUS, simulated weightlessness group.

PICRUSt was used to predict the metagenomic contribution of intestinal microbiota based on the information of OTUs against KEGG database at different hierarchical levels. At level 2, 39 gene families were identified, with the majority belonging to carbohydrate metabolism (10.39% in CON group, and 10.47% in SUS group), amino acid metabolism (9.86% in CON group, and 9.66 in SUS group), replication and repair (9.77% in CON group, and 9.56% in SUS group), membrane transport (9.42% in CON group, and 10.47% in SUS group) and energy metabolism (6.26% in CON group, and 5.89% in SUS group) (**Figure 7A**). At level 3, major pathways in both groups were related to transport system, DNA repair and recombination proteins, ribosome, purine and pyrimidine metabolism, peptidases and chromosome.

LEfSe was used to identify significant variations in the functional profile. At level 2, membrane transport was significantly higher, while genetic information processing, energy metabolism, metabolism of cofactors and vitamins were significantly lower in SUS group (LDA score > 2, **Figure 7B**). **Figure 7C** shows the biomarkers found at pathway level: 5 in SUS group and 8 in CON group. In detail, the pathways related with oxidative phosphorylation, cysteine and methionine metabolism, citrate cycle, riboflavin metabolism, folate biosynthesis, alanine, aspartate and glutamate metabolism were significantly decreased, with secretion system significantly increased in SUS group compared with CON group (LDA score > 2).

## Discussion

The present study employed a hindlimb unloading rats model to examine the response of intestinal mucosal barrier function of rats to simulated weightlessness. The hindlimb unloading model, also known as tail suspension, is a well-established ground-based approach simulating certain physiological effects during space flight (Morey-Holton and Globus, 2002; Globus and Morey-Holton, 2016). Under microgravity, the absence of hydrostatic pressure gradient results in redistribution of the blood from veins of the lower limbs to the chest and head, which are similar to that induced by tail suspension (Smith et al., 1997; Noskov, 2013). The insufficient supplies of blood and oxygen to intestine could disturb the normal functions of intestinal mucosal mechanical barrier (Xu et al., 2014). This study indicated the intestinal mucosa was seriously injured, and intestinal villi showed bizarre shape changes such as partial loss, sloughing and vacuolization in SUS group. It has been reported that the simulated weightlessness induced intestinal damage, as measured by histopathologic scores (Li et al., 2015). In particular, hindlimb unloading destroyed the structure of microvilli, and significantly reduced the surface area of microvilli (Chen et al., 2011). The real weightlessness during 7- to 18-days spaceflight may impair the morphology and function of colonic epithelial cells (Rabot et al., 2000). Furthermore, the present study demonstrated that the expression of pro-apoptotic protein Bax increased while the expression of anti-apoptotic protein Bcl-2 decreased in ileum of SUS group, indicating simulated weightlessness promoted apoptosis of intestinal epithelial cells.

Tight junction proteins are essential for integrity of intestinal barrier. The disruption of tight junction increases the permeability of intestinal barrier. In such situations, allergens, toxins and pathogens will pass the intestinal mucosal barrier, and induce gastrointestinal infection or systemic inflammatory responses (Chen et al., 2011). As the major tight junction proteins, claudin-1 and claudin-5 play crucial roles in maintaining epithelial barrier, down-regulation of these proteins leads to discontinuous tight junctions and barrier dysfunction (Zeissig et al., 2007; Wang et al., 2012). As a major component of adherens junctions, E-cadherin provides mechanical integrity and stability to intestinal epithelial lining (Schneider et al., 2010). This study showed that simulated weightlessness decreased the expression of occludin, claudin-1, claudin-5, and E-cadherin in ileum. To investigate the intestinal mucosal permeability, concentrations of DAO in serum were measured. It showed that simulated weightlessness significantly increased the DAO level in serum of rats. It was in line with the previous study, which reported that 21 days of tail-suspension reduced occludin and zonula occludens-1 (ZO-1) expressions, and improved the plasma concentrations of DAO and D-lactate (Chen et al., 2011). Those results elucidated simulated weightlessness could enhance the intestinal permeability through the damage of tight junctions, thus disrupt the barrier functions of intestinal mucosa.

The environmental stress in space can perturb the structure and composition of intestinal bacterial communities even during short spaceflights (Cervantes and Hong, 2016). As gastric emptying is highly influenced by the physiological status such as splanchnic blood flow and body position (Amidon et al., 1991), changes in fluid distribution and intestinal motility due to microgravity could slow down the gastric emptying and accelerate the intestinal transit time (Rabot et al., 2000). Meanwhile, stool frequency was negatively correlated with observed species and Chao 1 indices (Hadizadeh et al., 2017). The present study indicated that simulated weightlessness slightly increased the observed species and Chao 1 index of gut microbiota, which are in accordance with the previous study that reported the decreased microbial richness was associated with short intestinal transit time and osmotic diarrhea (Gorkiewicz et al., 2013).

Microgravity or simulated weightlessness has been shown to induce dysbiosis in intestinal microbiota (Smirnov and Lizko, 1987; Li et al., 2015; Ritchie et al., 2015). The present study indicated that hindlimb unloading increased proportion of *Bacteroidetes* and decreased *Firmicutes*-to-*Bacteroidetes* (F:B) ratio compared with the CON, which are in accordance with the change of intestinal microbiota during 13-days spaceflight (Ritchie et al., 2015). In addition, hindlimb unloading increased the proportion of *Treponema* in *Spirochaetes* phylum, while decreased that of *Ruminococcaceae unclassified, SMB53* and *Allobaculum* in Firmicutes phylum. Increased gut permeability has been shown to be associated with dysbiosis, such as a drastic decrease in abundance of Ruminoccoccaceae family (*Ruminococcus* and *Oscillibacter*) in alcohol-dependent subjects (Bull-Otterson et al., 2013; Leclercq et al., 2014), or a decrease in Firmicutes but an increase in Bacteroidetes in mice with chronic ethanol administration (Yan et al., 2011). Yeruva et al. (2016) reported that the improved expression of VE-cadherin is associated with high level of SMB53 genus in ileum of neonatal porcine model with breastfeeding. At the species level, the investigation has proved the increase of *Bacteroides* spp. and *Prevotella* spp. in intestinal mucosa with Ulcerative Colitis compared with healthy control subjects (Lucke et al., 2006). *S. termitidis* and *Clostridium* spp. were decreased in gut microbiota from children with celiac disease with reduction of ZO-1 expression (Cheng et al., 2013). In addition, *Bacteroides* ssp., *Prevotella* ssp. and *Treponema ssp.* can ferment xylene, xylose and carboxymethyl cellulose as carbohydrate metabolizers to produce high levels of short-chain fatty acids (SCFAs) (Obregon-Tito et al., 2015), while *Clostridium ssp.* were known to have the capability to produce butyrate with protective role against gut inflammation (Koh et al., 2016). The change of these bacteria proportion may explain the previous reports that 9-day spaceflight induced a significant increase of SCFAs concentration, but decreased the proportion of butyrate in cecal content of rats (Rabot et al., 2000). Butyrate is a major regulator of colonic cell proliferation and differentiation, its reduction may impair the morphology of intestinal epithelial cells and increase the permeability of intestinal mechanical barrier functions, which are often associated with digestive dysfunctions such as colonic irritability and diarrhea, or digestive pathologies such as ulcerative colitis and colonic neoplasia (Koh et al., 2016).

The results from PICRUSt indicated that simulated weightlessness increased the genes responsible for membrane transport, while decreased that involved in energy metabolism, and metabolism of cofactors and vitamins, suggesting that the microbiome in SUS group may have an decreased capacity for energy and micronutrient harvest (Ye et al., 2016). It may because of that the slowdown of gut peristalsis and acceleration of intestinal transit time decrease amounts of nutrients reaching the intestinal microbiota, thus improve their ability of membrane transport (Heyman-Lindén et al., 2016). Meanwhile, the increase of genes associated with xenobiotics biodegradation and metabolism such as xylene degradation and dioxin degradation, and replication and repair such as non-homologous end-joining were observed in SUS group, which were positively correlated with intestinal disruption, dysbiosis and inflammation (Rooks et al., 2014).

Recent data demonstrated the spaceflight is associated with immune system dysregulation (Crucian et al., 2014). During this process, dysbiosis of gut microbiota plays a crucial role. With the growth and stationary phases of bacterial growth, ET can be released from outer membrane of Gram-negative bacteria after cell disintegration and lysis (Liu et al., 2014). Interestingly, in the present study, the most increased species induced by simulated weightlessness belonging to the Bacteroidetes phylum are Gram-negative, while most decreased species belonging to the Firmicutes phylum are Gram-positive. Furthermore, one of the outcomes of gut dysbiosis induced by simulated weightlessness is a change in the expression of proteins of the enterocyte-tight junctions, resulting in increased gut permeability (Chen et al., 2011). Increased systemic ET due to increased Gram-negative bacteria such as *Bacteroides* and *Prevotella*, combined with disrupted gut barrier function can activate pattern-recognition receptors (PRRs), which may be linked to endotoxemia-induced metabolic inflammation (Yang et al., 2017). TLRs are transmembrane receptors that specifically recognize a variety of pathogen-associated molecular patterns from microbe (Velloso et al., 2015). As a best characterized PRR, TLR4 can be activated in response to the ET. The signaling pathway activated by TLR4 leads to recruitment of Myd88, which eventually causes NF-κB activation and inflammatory cytokines production (Fawkner-Corbett et al., 2017). Significantly increased of TLR4 expression in intestinal epithelial cells is associated with acute inflammation (Hausmann et al., 2002). Our results suggested that simulated weightlessness activated TLR4/MyD88/NF-κB signaling pathway, which was consistent with the increase of proinflammatory cytokines in serum such as IFN-γ and IL-4. Zhou et al. (2012) reported that hindlimb unloading markedly elevated circulating IFN-α, IL-6 and TNF-α in mice. The activation of NF-κB could potentially impact health that appeared to be always negative (Zhang et al., 2017). Moreover, this study showed the increased expression of TLR4/MyD88/NF-κB signaling proteins induced by simulated weightlessness was correlated with the damage of the morphology and structure of intestinal mucosa, and the decreased expression level of occludin, claudin-1 and claudin-5. A recent study reported that the microbiota of TLR4 overexpressing mice were found to be transmissible and exacerbated dextran sulfate sodium (DSS) induced colitis. The increased expression of TLR4 was associated with an impaired barrier, increased intestinal permeability, and altered expression of anti-microbial peptides (Dheer et al., 2016). Thus, simulated weightlessness-induced activation of TLR4/MyD88/NF-κB may influence the tight junction complexes and eventually cause damage to the intestinal barrier that results in bacterial translocation. Previous studies have demonstrated that hindlimb unloading could affect the ability to fight bacterial infections, which was due to impaired barrier integrity of the gastrointestinal tract (Belay et al., 2002). In addition, TLR4 signaling has been shown to affect the intestinal flora (Frosali et al., 2015). Regulation of the microbiota by TLR4 appears to be attributable to differentiation of goblet cells (Sodhi et al., 2012), and alterations in gastrointestinal motility that drives clearance of pathogens and maintenance of commensal populations (Anitha et al., 2012). These results suggested that TLR4/MyD88/NF-κB signaling pathway is associated with the damaged intestinal barrier function and dysbiosis of microbiota induced by simulated weightlessness.

Studies have demonstrated a decrease in total WBC count, LYM, MON, and eosinophils, and a slight increase in NEU in rats flown on a 9 and 14-day mission (Allebban et al., 1994; Ichiki et al., 1996). This study indicated that simulated weightlessness significantly decreased the concentration of WBC and LYM, while markedly increased the relative percentage of NEU in peripheral blood. In the previous studies, decline in lymphocyte growth with increased apoptosis, chromosomal aberrations, inhibited locomotion, and altered cytokine production have been described under altered gravity conditions (Cervantes and Hong, 2016). Neutrophilia has also been found in astronauts returning from spaceflight (Stowe et al., 1999). Li et al. (2015) reported increased neutrophils migration into the colon tissue in hindlimb unloading model, which may work together with elevated IL-1β to perpetuate the inflammatory environment and subsequently increase the susceptibility to DSS-induced colitis.

During the real space flight, the astronauts are placed in a multistressor environment. Beside the extreme environment, such as microgravity and cosmic radiation, they also suffer from stress all the time. However, for human physiology studies under space flight, it is not easy to separate each signal environmental factor and distinguish its physiological influence. The influence of hindlimb unloading on intestinal barrier and microbiota may simply be induced by stress (Globus and Morey-Holton, 2016). Stress is also known to be able to alter the expression of proinflammatory cytokines, and damage intestinal barrier functions (Ferrier et al., 2003; Yang et al., 2006). Therefore, the main limitation of this study is the lack of stress CON. Response of intestinal mucosal barrier functions to hindlimb unloading compared with stress CON still requires further investigation.

## Conclusion

**FIGURE 8:**
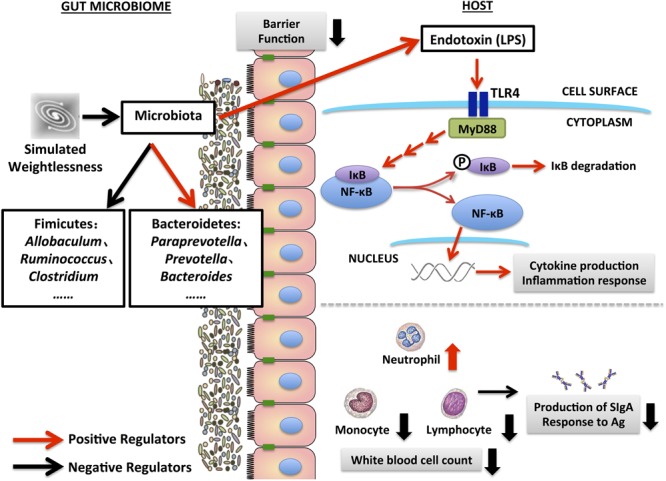
Schematic diagram for the disruption of intestinal mucosal barrier functions induced by simulated weightlessness.

The present study indicated that one of the simulated weightlessness analogs, the hindlimb unloading rats model, could induce pathological changes in intestinal mechanical barriers, including damage to the intestinal villi, down-regulation of tight junction proteins expression, induction of apoptosis, thus improvement of intestinal permeability. Together with the expansion of Bacteroidetes, decrease of Firmicutes, and changes of the certain intestinal bacteria, such as *Allobaculum, Ruminococcus, Clostridium, Paraprevotella, Prevotella*, and *Bacteroides*, it increased the level of systemic ET, which contributed to the disrupted intestinal immune homeostasis through the activation of TLR4/MyD88/NF-κB signaling pathway, increase of proinflammatory cytokines, and decrease of secretory immunoglobulin A level (as summarize in **Figure 8**). The current findings emphasize the necessity of monitoring and regulating astronauts’ intestinal health during real space flights. Results also highlight the importance of intestinal mucosal barrier functions in the health status of astronauts. In our future work, the mechanisms by which the real weightlessness interacts with intestinal mucosal barrier functions will be elucidated, which will provide clues for developing rational avenues and countermeasures to prevent breakdowns in intestinal homeostasis of crewmembers.

## Author Contributions

MJ and HY conceived the project and designed the experiments. MJ and HZ performed the experiments. MJ, KZ, CX, and DS analyzed the data. MJ and HY wrote the manuscript, with KZ, QH, and JS providing text and thorough editing. All authors helped with data interpretation, discussion of the results, and review of the manuscript.

## Conflict of Interest Statement

The authors declare that the research was conducted in the absence of any commercial or financial relationships that could be construed as a potential conflict of interest.

## Footnotes

1 https://huttenhower.sph.harvard.edu/galaxy/

2 http://huttenhower.sph.harvard.edu/galaxy/

3 http://picrust.github.io/picrust

## Supplementary Material

FIGURE S1 Expression of Bax and Bcl-2 in ileum by immunohistochemistry assay. CON, control group; SUS, simulated weightlessness group.

FIGURE S2 Expression pattern of claudin-1 and E-cadherin in ileum by immunohistochemistry assay. CON, control group; SUS, simulated weightlessness group.
