# Supplementary figures and images for: Responses of Intestinal Mucosal Barrier Functions of Rats to Simulated Weightlessness
Source: Front Physiol. 2018 Jun 14;9:729. doi: 10.3389/fphys.2018.00729 (PMC6011188; doi:10.3389/fphys.2018.00729)

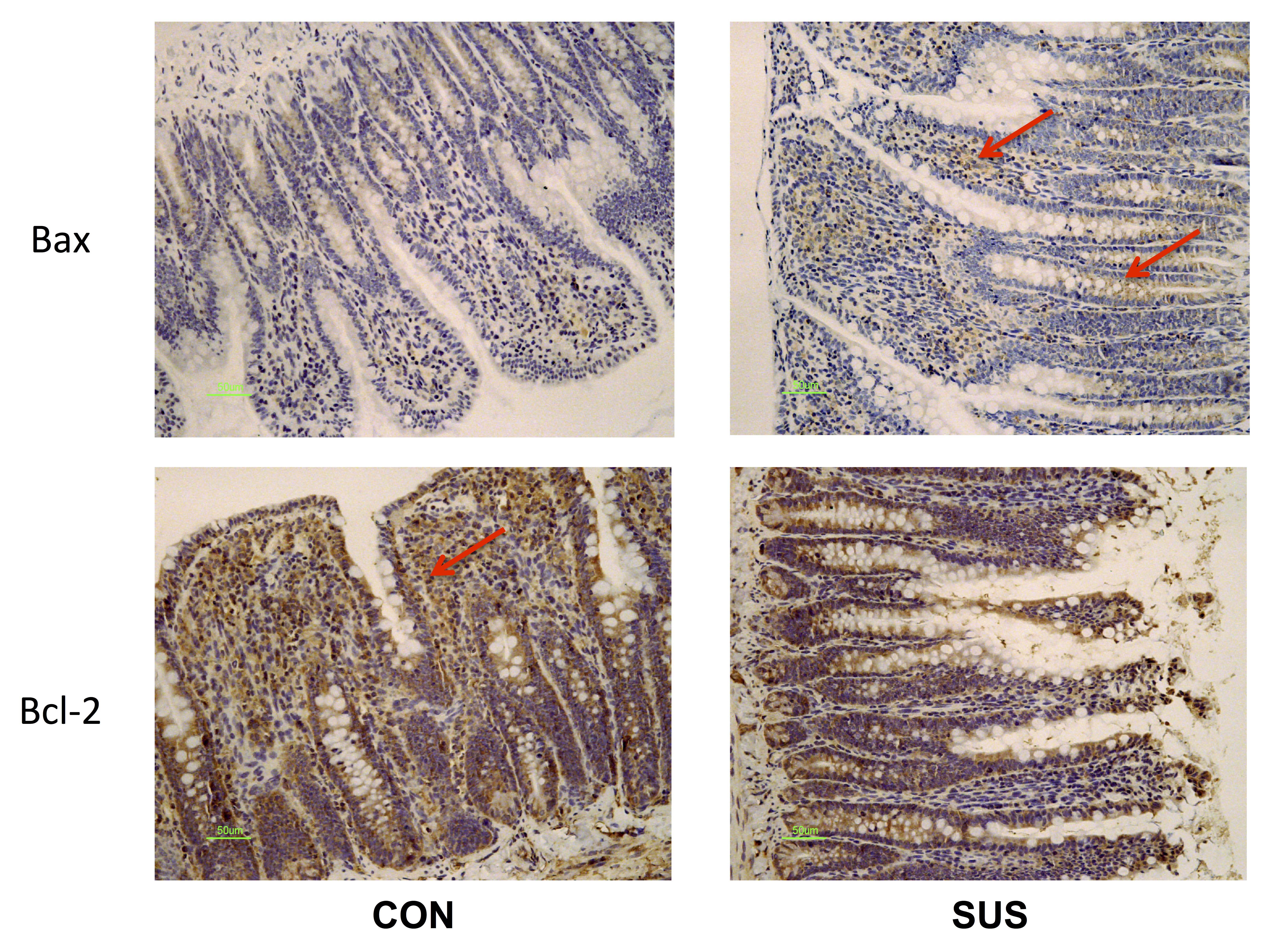

Supplement: FIGURE S1 — Expression of Bax and Bcl-2 in ileum by immunohistochemistry assay. CON, control group; SUS, simulated weightlessness group. [file Image_1.JPEG]

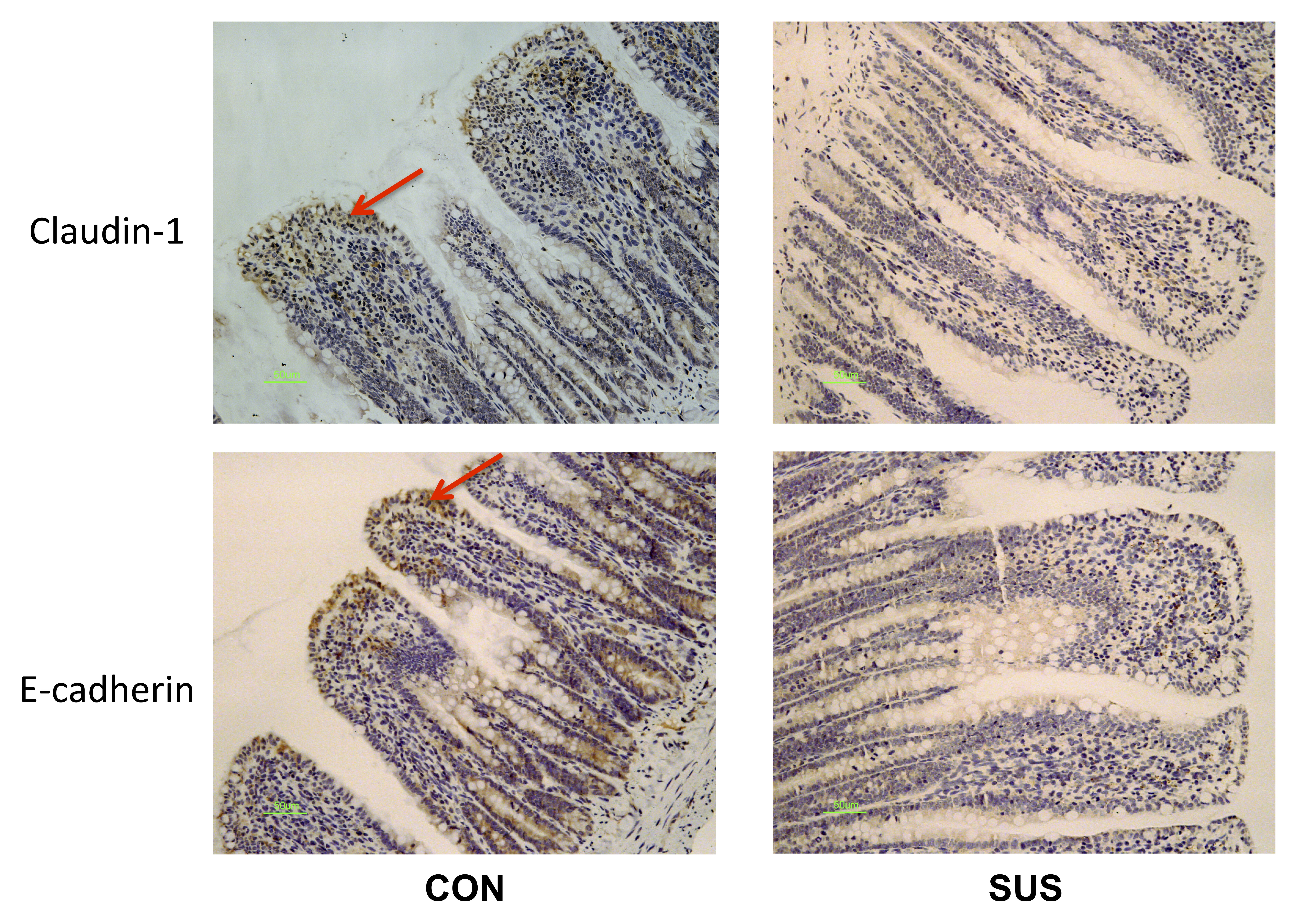

Supplement: FIGURE S2 — Expression pattern of claudin-1 and E-cadherin in ileum by immunohistochemistry assay. CON, control group; SUS, simulated weightlessness group. [file Image_2.JPEG]
